# Fat-forming variant of solitary fibrous tumor (lipomatous hemangiopericytoma) of the chest wall: a rare tumor in a rare location

**DOI:** 10.1186/1749-8090-8-S1-P129

**Published:** 2013-09-11

**Authors:** C Foroulis, A Kleontas, C Poulios, V Tzioufa, K Anastasiadis

**Affiliations:** 1 Department of Cardiothoracic Surgery, AHEPA University Hospital, Thessaloniki, Greece; 2 Department of Pathology, AHEPA University Hospital, Thessaloniki, Greece

## Backround

Lipomatous hemangiopericytoma (LHPC) is a very rare tumor with uncertain biologic behavior. This report handles with the clinical, radiological and histological features of the 1st case to date of LHPC, occurring in the thoracic inlet and the surgical technique is described.

## Methods

A 18-year-old, non smoking, male, patient, with no significant medical history, was referred with a 6-month history of right-sided chest pain. On physical exam, an oval-shaped mass was palpated in the right supraclavicular area. Imaging findings revealed a well-circumscribed, inhomogeneous tumor in the posterior compartment of the right thoracic inlet, with dimensions of 5.3 X 8.9 X 10.8 cm. The tumor seemed to compress the adjacent structures, but not clearly to infiltrate them, while it’s lower limit reaching the 4th intercostal space.

## Results

An incisional biopsy was performed, resulting in the early diagnosis of LHPC. A right upraclavicular incision was performed at a second time to access the tumor. The clavicle was transected at its middle in order to enlarge the surgical field, allowing that way excision of the tumor with intact capsule, through a combination of sharp and blunt dissection. A lot of feeding vessels were clipped or ligated during dissection within the thoracic inlet. The patient had an uneventful short postoperative course. The diagnosis of LHPC was confirmed by two more laboratories. He underwent adjuvant radiation therapy in the right thoracic inlet and supraclavicular area, to reduce the risk of recurrence, because the tumor was violated during the incisional biopsy.

## Conclusion

The thoracic inlet is one more location for the very rare LHPC. The anatomic topography of the mass requires special maneuvers to achieve safe removal of tumor with intact capsule, such as transection of the clavicle. Adjuvant radiation should be discussed within the multidisciplinary oncology team, as the global experience with these rare tumors is limited.

